# MRI Imaging of Double Pituitary Microadenoma: A Rare Preoperative Diagnosis

**DOI:** 10.7759/cureus.24100

**Published:** 2022-04-13

**Authors:** Pratik J Bhansali, Bhushita Lakhkar, Rajasbala P Dhande, Bhushan Lakhkar

**Affiliations:** 1 Radiodiagnosis, Jawaharlal Nehru Medical College, Datta Meghe Institute of Medical Sciences (Deemed To Be University), Wardha, IND

**Keywords:** benign tumors, monoclonal neoplasms, pituitary adenomas, double adenomas, adenoma

## Abstract

Pituitary adenomas are benign, single, monoclonal slow-growing neoplasms usually related to chemical overproduction. A pituitary adenoma is the third most common intracranial tumor, with the first two being glioma and meningioma. Double pituitary adenoma is an infrequently occurring case and is characterized as occurrence of two adenomas in the single pituitary gland, both having typical immunohistochemical and histopathological highlights. In most of the cases, pituitary adenomas occurring as double adenomas are associated with Cushing's disease. It is further categorized into contiguous and clearly distinct types. Clearly distinct tumors are recognized on neuroradiological imaging. We present imaging findings of a rare case of a double pituitary microadenoma. Early diagnosis of such a rare condition is important and prevents further consequences.

## Introduction

Pituitary adenomas are benign, single, monoclonal slow-growing neoplasms usually related to chemical overproduction [1]. Double pituitary adenomas are characterized as the occurrence of two adenomas in the single pituitary gland, both having typical immunohistochemical and histopathological highlights [2]. It is further categorized into contiguous and clearly distinct types. Clearly distinct tumors are recognized on neuroradiological imaging [3]. We present a case report of a 21-year-old female presenting with a complaint of amenorrhea, which, on further evaluation, was found to be a case of double pituitary microadenoma, which is a rare finding on neuroimaging.

## Case presentation

**Figure 1:**
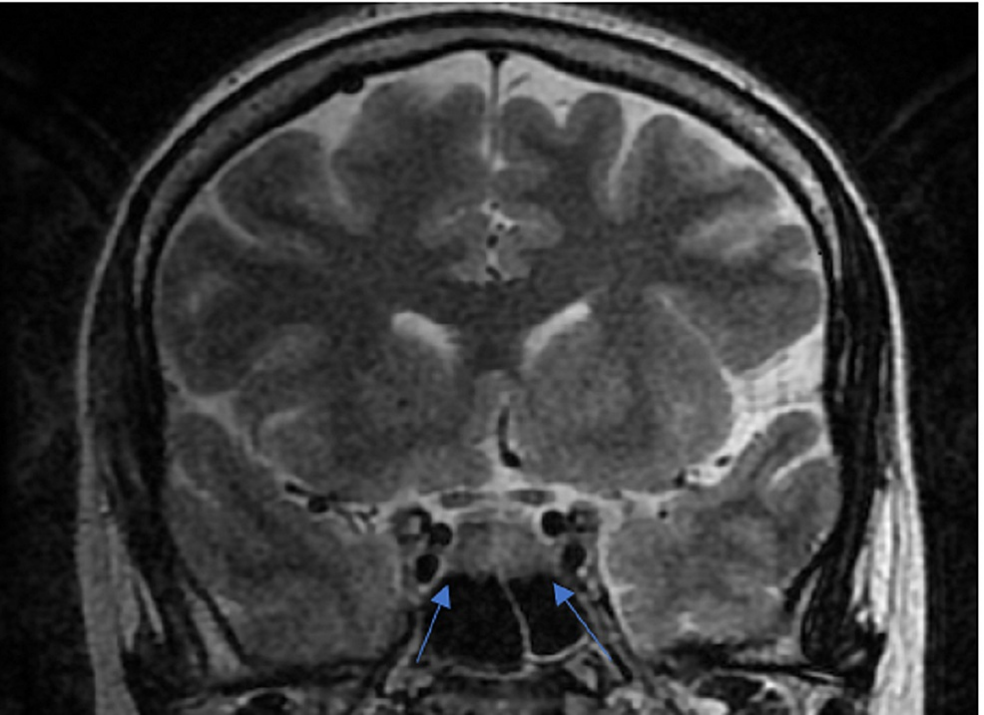
Coronal T2-weighted image showing two isointense lesions (arrow), with hyperintense normal pituitary tissue separating both the lesions.

**Figure 2:**
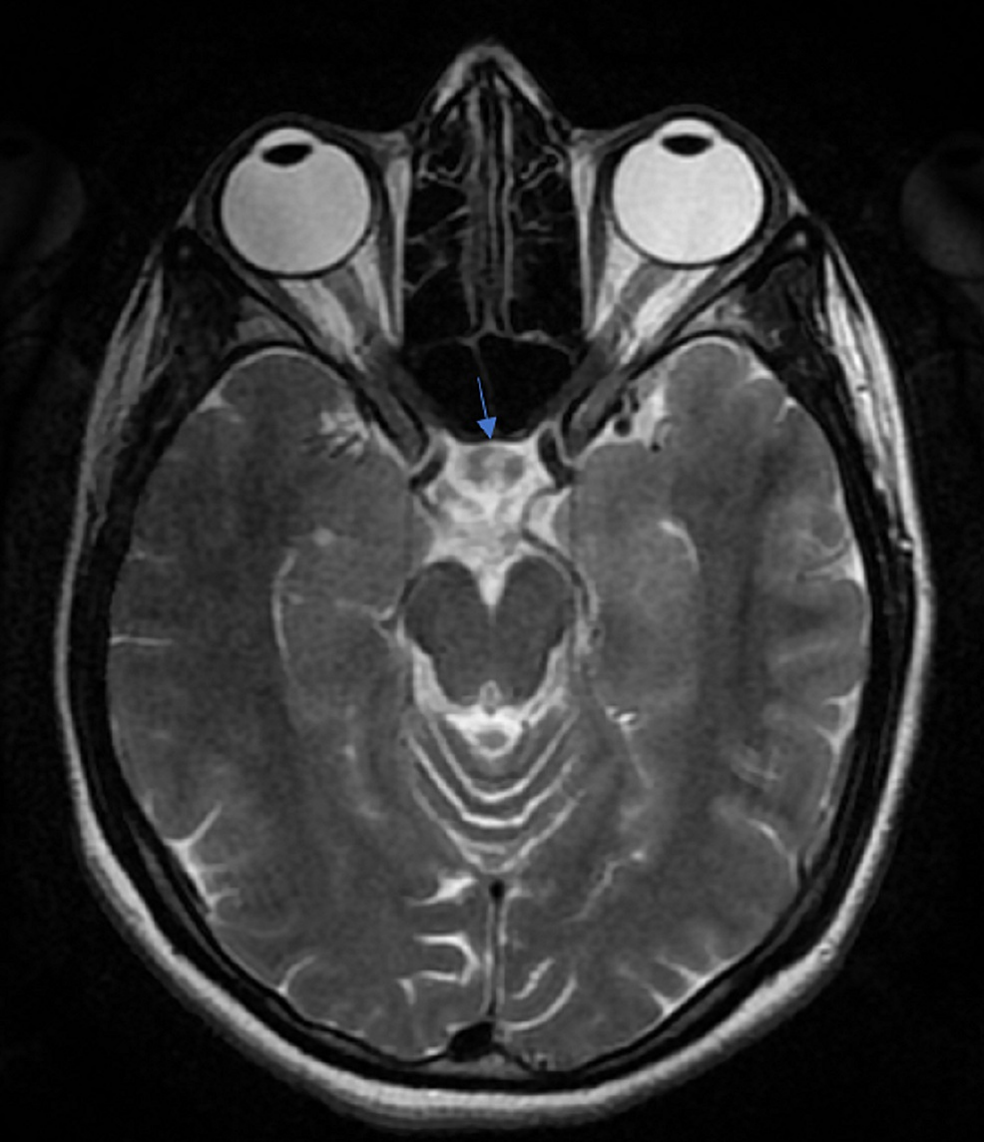
T2-weighted axial image showing two hypointense lesions, with hyperintense normal pituitary tissue between them (arrow).

**Figure 3:**
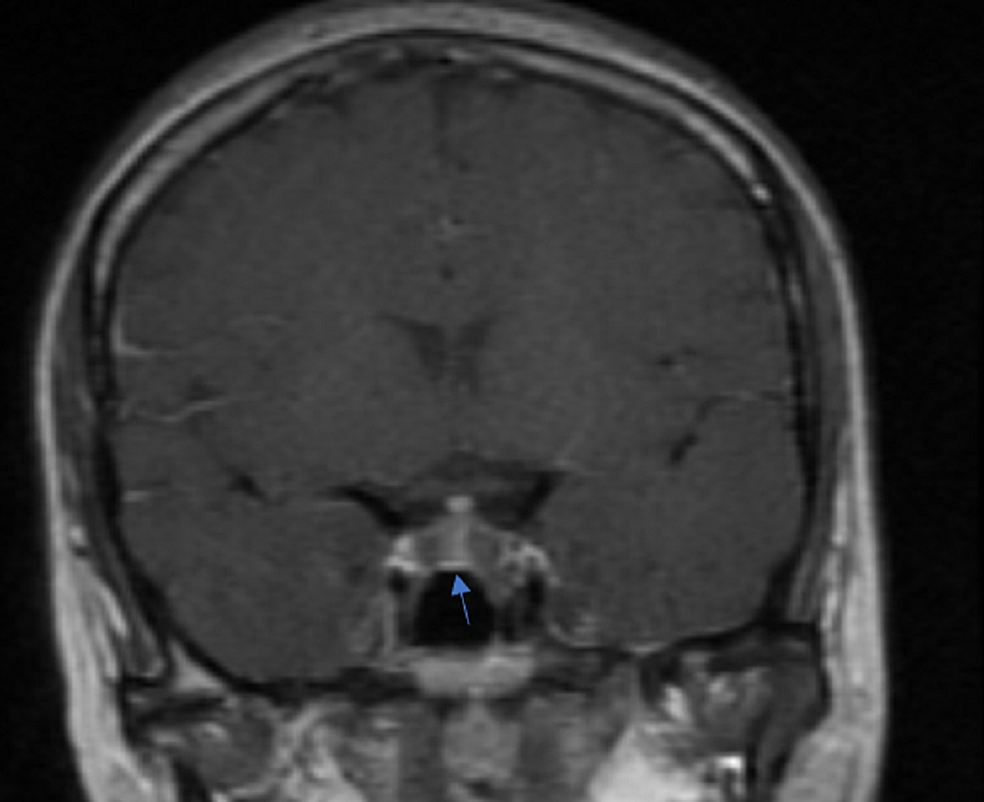
Postcontrast coronal section image shows two non-enhancing lesions within the pituitary gland with enhancing normal pituitary tissue separating the two lesions (arrow).

A 21-year-old female presented to our hospital with complaints of amenorrhea for the last four months and weight loss. On examination, the patient's general condition was fair, and vitals were normal. On systemic examination, cardiovascular, respiratory, and abdominal systems were normal. Neurologically, the patient was conscious and well oriented, and had no neurological deficit. On cranial nerve examination, there was normal visual acuity and normal visual field, and other cranial nerves were normal. On ultrasound, the patient was diagnosed with polycystic ovary disease, and on further hormonal investigations, the thyroid-stimulating hormone (TSH) level was 0.01 µIU/mL, which was below normal, and adrenocorticotropic hormone (ACTH) level was raised. For further evaluation, a brain MRI with gadolinium contrast was performed, which revealed two tiny altered signal intensity lesions in the anterior lobe of the pituitary gland. Both the lesion appeared isointense on T2- and T1-weighted images (Figures 1, 2) and hypointense on FLAIR (fluid-attenuated inversion recovery). This lesion showed no restriction on DWI (diffusion-weighted Imaging) and no blooming on GRE (gradient- recalled echo) sequence. On injecting gadolinium contrast intravenously at a dosage of 0.01 mmol/kg, both the lesion showed no contrast enhancement (Figure 3). Both the lesions measured 5.7 x 5.4mm and 5.8 x 5.7mm, respectively. The remnant pituitary gland showed normal enhancement. There was no involvement of cavernous sinus and no compression of optic chiasma and surrounding tissue. Hence, the patient did not have any pressure-related or ophthalmic symptoms. Thus, a diagnosis of double pituitary microadenoma was made. As the lesions were not compressing the adjacent structures, and the patient was free from pressure-related symptoms, she was managed conservatively with hormonal therapy. On follow-up after three weeks, the patient was asymptomatic and doing well.

## Discussion

Double pituitary adenoma is an infrequently occurring tumor, with an incidence rate of 0.9% in random pituitary autopsy samples [4]. Their prevalence rate ranges from 0.25 to 2.6% of postoperated pituitary adenoma specimen [5-6]. As the use of high-field MRI has increased in the recent years for suspected pituitary pathologies, the preoperative detection of double pituitary adenoma HAS also increased, which is aided by cytological analysis [7]. Majority of the cases reported are the findings on the autopsy samples, and we present a case report of young female as a preoperative finding. Various theories have been put forward to delineate the pathogenesis of double pituitary adenomas. The first hypothesis explains unplanned monoclonal extension of two unmistakable hereditarily transformed types of pituitary cells and is upheld with help of particular tumor containers at microdissection of careful examination. The transdifferentiation hypothesis depends on the capacity of pituitary adenoma cells of one type to change or transdifferentiate into other types of cells. The transdifferentiation hypothesis is aided with help of transcription factor expression and hereditary profiling of double pituitary adenomas [8].

Pituitary adenomas are segregated on the basis of size: if measuring ≤ 10 mm, it is considered as microadenoma, and if > 10 mm, it is considered as macroadenoma [9]. Sumida et al. described pituitary adenoma on MRI appearing as isointense on T1-weighted images with no enhancement after contrast imaging [10]. A study conducted by Oner et al. found two non-enhancing foci in the gland separated by normal gland tissue [11]. In the study by Zieliński et al., on preoperative imaging there were two microadenomas separated by normal pituitary between the two tumors. These imaging findings were confirmed on immunohistochemistry performed postoperatively, which revealed two distinct types of adenoma. Preoperative MRI imaging has a great role in in identification of dual adenomas as its preoperative diagnosis may prevent chances of relapse and surgical failure [8].

In our case, findings on MRI imaging are similar to those described by Sumida et al., suggesting that both the lesions are pituitary microadenomas. In comparison with the study by Oner et al. and Zieliński et al., we can confirm the presence of double pituitary microadenoma.

## Conclusions

Double pituitary microadenoma is a rare tumor, and the majority of the patients are asymptomatic at an early age. Hence, they are very difficult to diagnose clinically. Any hormonal imbalance related symptoms must be further evaluated for their cause. It is important to diagnose early as it can present with vision disorder due to compression over optic chiasma or severe headache due to compression over cavernous sinus. MRI imaging provides its early diagnosis with a correlation with hormonal levels.
